# Small intestinal injury in NSAID users suffering from rheumatoid arthritis or osteoarthritis

**DOI:** 10.1007/s00296-016-3552-x

**Published:** 2016-08-22

**Authors:** Ilja Tachecí, Petr Bradna, Tomáš Douda, Drahomíra Baštecká, Marcela Kopáčová, Stanislav Rejchrt, Martin Lutonský, Tomáš Soukup, Jan Bureš

**Affiliations:** 1 Second Department of Internal Medicine – Gastroenterology, Faculty of Medicine at Hradec Králové, University Hospital, Charles University in Prague, Sokolská 581, 50003 Hradec Králové, Czech Republic; 2 Department of Orthopaedic Surgery, Faculty of Medicine at Hradec Králové, University Hospital, Charles University in Prague, Sokolská 581, 50003 Hradec Králové, Czech Republic

**Keywords:** Non-steroidal anti-inflammatory drug, Small bowel, Wireless capsule endoscopy, Enteropathy, Rheumatoid arthritis, Osteoarthritis

## Abstract

The goal of this prospective study was to assess non-steroidal anti-inflammatory drug (NSAID)-induced enteropathy in patients with rheumatoid arthritis (RA) or osteoarthritis (OA) by means of non-invasive wireless capsule enteroscopy. A total of 143 patients (74 with RA, 69 with OA) treated with NSAIDs (>1 month) and 42 healthy volunteers were included. All subjects underwent capsule endoscopy, laboratory tests and filled in questionnaires. The severity of small bowel injury was graded as: mild (red spots or sporadic erosions), moderate (10–20 erosions) or severe (>20 erosions or ulcers). Capsule endoscopy identified small bowel lesions in 44.8 % of patients (mild 36.4 %, moderate 3.5 % and severe in 4.9 %). Mild non-specific lesions were found in 11.9 % healthy volunteers. There was a significantly higher prevalence of enteropathy in RA (56.8 %) compared to OA (31.9 %, *p* < 0.01). A significant difference between NSAID users (RA and OA) with and without enteropathy was observed in erythrocytes (*p* < 0.01), the leucocyte count (*p* < 0.05), haemoglobin (*p* < 0.05), haematocrit (*p* < 0.05), serum albumin (*p* < 0.01) and erythrocyte sedimentation rate (*p* < 0.05). No relationship was found between enteropathy and dyspepsia, gender or age. NSAID therapy is associated with a significant risk of small bowel injury. The risk is significantly higher in RA patients suggesting a possible influence of the underlying disease.

*Trial registration number*: DRKS00004940.

## Introduction

Non-steroidal anti-inflammatory drugs (NSAIDs) are some of the most commonly prescribed drugs all over the world [1]. Their use can be limited by adverse side effects to the gastrointestinal tract. NSAID-induced small intestinal injury was underestimated in the past because of lack of suitable diagnostic tools. Recently, NSAID-induced enteropathy has paid more attention to this issue owing to wireless capsule and deep enteroscopy being available [2].

NSAID-induced enteropathy might be asymptomatic or can be presented with increased small intestinal permeability, bleeding, erosions, ulcers, bowel perforation, diaphragm-like strictures, and/or jejunal and ileal dysfunction.

Wireless capsule endoscopy is a useful method for non-invasive diagnosis of enteropathy. Small bowel lesions were revealed in 26–88 % COX non-selective and 6–50 % COX-2 selective NSAID users [3–8].

### Objective

 The goal of this prospective study was to assess NSAID-induced enteropathy in RA or OA by means of non-invasive wireless capsule enteroscopy.

## Methods

### Patients

The project was designed as a prospective and endoscopist-blinded study. Inclusion criteria comprised adult patients (>18 years) treated for RA or OA by NSAIDs (>1 month) and adult healthy volunteers without any medical therapy. RA fulfilled the 1987 criteria of the American College of Rheumatology [9]. OA was confirmed by X-ray.

Exclusion criteria were gastro-oesophageal reflux disease, peptic ulcer disease, all known small bowel diseases, including inflammatory bowel disease and pregnancy.

A total of 143 NSAID users in RA (74 patients, 57 women, mean age 52, median 53 years) or OA (69 patients, 44 women, mean age 67, median 67 years) and 42 control healthy volunteers (29 women, mean age 43, median 42 years) were enrolled. All patients underwent capsule endoscopy, completed a questionnaire focused on history and clinical data, and laboratory tests (blood count, Coombs test, iron-binding capacity, serum iron, ferritin, albumin, prealbumin, C-reactive protein and erythrocyte sedimentation rate). Stools were tested for the *Helicobacter pylori* antigen and for faecal occult bleeding.

### Capsule endoscopy

A wireless capsule endoscopy system was used (EndoCapsule, Olympus) after 12-h fasting. Fluids were allowed 2 h after ingestion of the capsule endoscope, followed by a light meal 4 h later. Capsule position was verified 2 h after ingestion by means of a real-time viewer. Gastroscopy-assisted insertion of the capsule endoscope through the pylorus was performed in three cases of capsule persistence in the stomach. The capsule endoscopy investigator (I.T.) was experienced in enteroscopy and blinded to all data.

Type and localisation of small bowel lesions, other abnormal findings and transit times were evaluated. Endoscopy findings were described as red spots (reddish area of mucosa), erosions (superficial destruction of mucosa), denuded areas (mucosal surface without villi), aphthous lesions (a mucosal break with a pale centre and reddish halo) and ulcers (a local defect in lining or excavation of the mucosa surface with its base covered with fibrin). Findings were classified into three grades as mild (multiple red spots or <10 erosions and/or aphthous lesions), moderate (10–20 erosions or aphthous lesions) and severe (>20 erosions or aphthous lesions, ulcers, stenosis and/or bleeding). Localisation of lesions was roughly estimated according to transit time and actual capsule position on the screen of computer workstation. Small intestinal visibility in different segments of the bowel was scored.

### Statistics

Qualitative data were compared using contingency tables, a Chi-squared test for independence and Goodman–Kruskal gamma test for trend evaluation. We used two-sample *t* test, Mann–Whitney test, one-factor analysis of variance and Kruskal–Wallis test. Testing was performed on a significance level of 5 %.

## Results

### Endoscopy findings

All participants underwent capsule endoscopy without any complications. Complete small bowel investigation was performed in 99 % of all procedures in NSAID users and in all healthy volunteers. Excellent small bowel visibility was achieved in 142 of 185 (77 %) capsule endoscopies; there was some bowel content in the distal ileum in 43/185 (23 %).

**Fig. 1:**
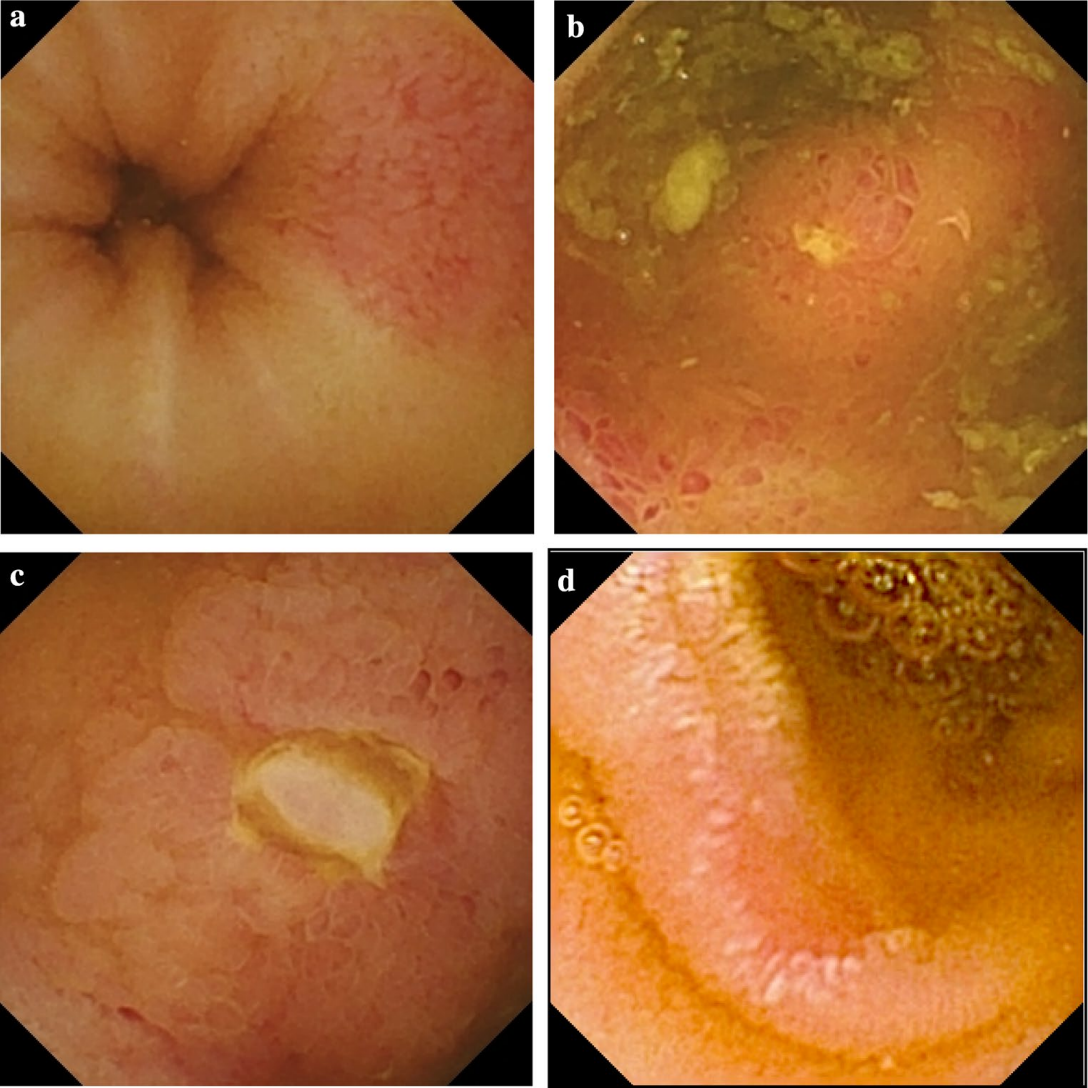
NSAID-induced enteropathy grading. **a** mild grade: red spot in jejunum, **b** moderate grade: multiple erosions in distal jejunum, **c** severe grade: ulceration in ileum, **d** severe grade: jejunal ulcer

NSAID-induced enteropathy was observed in 64/143 NSAID users (44.8 %). Mild non-specific findings (sporadic red spots) were identified in 5 (11.9 %) healthy volunteers (*p* < 0.001). A statistically significant difference between the RA and OA was identified in the prevalence (56.8 vs. 31.9 %, *p* < 0.01) and severity of enteropathy (*p* < 0.01). The small intestinal findings observed included red spots or mucosal erythema, erosions, aphthous lesions and ulcers (Fig. 1). No strictures, bleeding or denuded areas were revealed. Other small intestinal findings, not associated with NSAIDs, were: lymphangiectasias, phlebectasias, xanthomas and lipoma. NSAID-induced gastropathy was observed in 33.8 % RA and 21.7 % OA.

There was no difference in gastric emptying time and small bowel transit time in NSAID users according to the presence and severity of enteropathy (data not displayed).

### Clinical data

In the RA group, the mean age of RA diagnosis was 42 years. Rheumatoid factors were positive in 54 cases (73 %), and RA was classified as grade I (according to Steinbrocker’s classification) in 5, grade II in 29, grade III in 31 and grade IV in nine patients. The average disease activity score (DAS 28) was 3.46 ± 1.49 (median 3.28). No statistically significant difference in the DAS 28 score and RA grading according to presence and the severity of identified enteropathy was observed.

All patients were treated with NSAIDs, most for several years: >1 year 70 (95 %) RA and 62 (90 %) OA. There was no difference in the prevalence of identified enteropathy in the context of COX selectivity or duration of use of NSAIDs, DAS 28 score and/or age and gender and/or either in RA or OA (data not displayed). NSAID users with concomitant acetylsalicylic acid or systemic glucocorticoid therapy had a higher rate of enteropathy in comparison with the remaining patients (*p* = 0.029, *p* = 0.003). Other medical treatment (e.g. biologic therapy, disease-modifying antirheumatic drugs, proton pump inhibitors, warfarin, bisphosphonates and others) did not influence the prevalence of enteropathy identified by capsule enteroscopy.

Dyspepsia, defined as any type of abdominal discomfort, including nausea, vomiting, abdominal fullness, pain, constipation and/or diarrhoea, was identified in 28 (38 %) RA and in 52 (75 %) OA (*p* < 0.01).

### Laboratory tests

A statistically significant difference between patients with and without enteropathy was found in erythrocytes (*p* = 0.002), haemoglobin (*p* = 0.015), haematocrit (*p* = 0.044), leucocytes (*p* = 0.023), erythrocyte sedimentation rate after 1 h (*p* = 0.036), serum albumin (*p* = 0.007) and positive faecal occult blood test (*p* = 0.010). There was no difference in *Helicobacter pylori* prevalence (20 patients, 14 %). Anaemia was more common in RA (31 patients; 42 %) compared to OA (4; 6 %).

## Discussion

Our study found a high prevalence of enteropathy in chronic NSAID users and provided important new original data. Capsule endoscopy identified small intestinal lesions in 44.8 % patients, but only a few lesions were assessed as moderate (3.5 %) or severe (4.9 %). That is much less than previously reported [7, 8, 10].

The most important finding was a significantly higher prevalence and severity of enteropathy in RA patients compared to OA. The influence of principal immunopathology, playing a crucial role in RA, might be a possible explanation. Similarly, the prospective data from the arthritis, rheumatism and ageing medical information system (ARAMIS) showed a higher risk of serious gastrointestinal complications of NSAIDs in RA when compared to OA (13 vs. 7.3 per 1000 patients per year) [11–13]. We are fully aware of the possible limits; thus, our findings must be interpreted with caution. Numbers of patients in our study are relatively low. There are other possible confounding factors such as different types and daily and cumulative doses of NSAID.

Although lower prevalence of small intestinal damage in COX-2 selective NSAID users was repeatedly demonstrated in comparison with non-selective NSAID users [3], we did not observe this phenomenon in our study. An explanation for this may be the relatively low proportion of COX-2 selective NSAID users (RA 13 %, OA 9 %) and other mechanisms of small bowel toxicity (chemical structure and local effect, metabolism, rates of enterohepatic circulation, etc). We proved a higher risk of enteropathy in patients with concomitant acetylsalicylic acid and/or glucocorticoids (but not with other medical therapy). A substantial number of our patients were also treated with proton pump inhibitors (80 % RA, 35 % OA). Recent studies found that proton pump inhibitors can exacerbate NSAID-induced small intestinal injury by inducing bacterial dysbiosis of the small bowel [14, 15]. We were not able to confirm these findings.

Surprisingly, mild non-specific mucosal changes at capsule endoscopy were also found in 12 % of the healthy volunteers. All of our control subjects were healthcare professionals at our University Hospital, available for a subsequent 3-year follow-up. All remained clinically healthy, symptom-free and developed no illness. Similar findings of mild mucosal changes during capsule endoscopy were also found in 7–41 % of control subjects in other previously published studies [3–5, 10]. It is necessary to emphasise this fact and to interpret these mild non-specific findings (like sporadic red spots) with caution.

Dyspepsia was significantly more frequently recorded in OA (75 %) compared with RA (38 %) in our study, but there was no association with small intestinal findings during capsule endoscopy.

## Conclusions

Wireless capsule enteroscopy is an important non-invasive diagnostic tool to diagnose NSAID-induced injury to the small bowel. Small intestinal lesions were identified in 44.8 % of patients in our current study, mostly mild (36.4 %), less frequently moderate (3.5 %) or severe (4.9 %). These lesions were more frequently found in RA (56.8 %) compared to OA (31.9 %). No laboratory marker and/or clinical data can be used as a diagnostic or prognostic factor.
